# A Review on the Electrochemically Self-organized Titania Nanotube Arrays: Synthesis, Modifications, and Biomedical Applications

**DOI:** 10.1186/s11671-018-2597-z

**Published:** 2018-06-28

**Authors:** Yu Fu, Anchun Mo

**Affiliations:** State Key Laboratory of Oral Diseases, Department of Implantology, West China Hospital of Stomatology, Sichuan University, Chengdu, 610041 China

**Keywords:** Titania nanotubes, Biomedicine, Electrochemical anodization, Modifications

## Abstract

Titania nanotubes grown by anodic oxidation have intrigued the material science community by its many unique and potential properties, and the synthesis of technology is merging to its mature stage. The present review will focus on TiO_2_ nanotubes grown by self-organized electrochemical anodization from Ti metal substrate, which critically highlights the synthesis of this type of self-organized titania nanotube layers and the means to influence the size, shape, the degree of order, and crystallized phases via adjusting the anodization parameters and the subsequent thermal annealing. The relationship between dimensions and properties of the anodic TiO_2_ nanotube arrays will be presented. The latest progress and significance of the research on formation mechanism of anodic TiO_2_ nanotubes are briefly discussed. Besides, we will show the most promising applications reported recently in biomedical directions and modifications carried out by doping, surface modification, and thermal annealing toward improving the properties of anodically formed TiO_2_ nanotubes. At last, some unsolved issues and possible future directions of this field are indicated.

## Introduction

Since the beginning of the twentieth century, titanium dioxide (TiO_2_) has been used as commercial production in sun-blockers, paints, sensors, photocatalysis, solar cells, electrochromic devices, drug delivery, etc. [1–7]. The phenomenon that TiO_2_ can produce the photogenerated electron-hole pairs under lighting irradiation can help split water into oxygen and hydrogen, benefiting to solve the energy crisis in the future as the most potential fuel. Fujishima and his co-workers first reported the photocatalytic water splitting on a TiO_2_ electrode under ultraviolet (UV) light [8–10], and since then, titanium dioxide has become one of the most studied compounds in material science. Among all transition metal oxides, it presents a broad range of functional properties like chemical inertness, corrosion-resistance, and stability, especially the improvement of biocompatibility [11], and electrical and optical properties [1]. Ever since Iijima discovered carbon nanotubes in 1991 [12], showing a unique combination between the shape and functionality, where properties can be influenced directly by the geometry, enormous efforts have been made in the field of nanotechnology basically in chemical, physical, and biomedical material science.

**Fig. 1:**
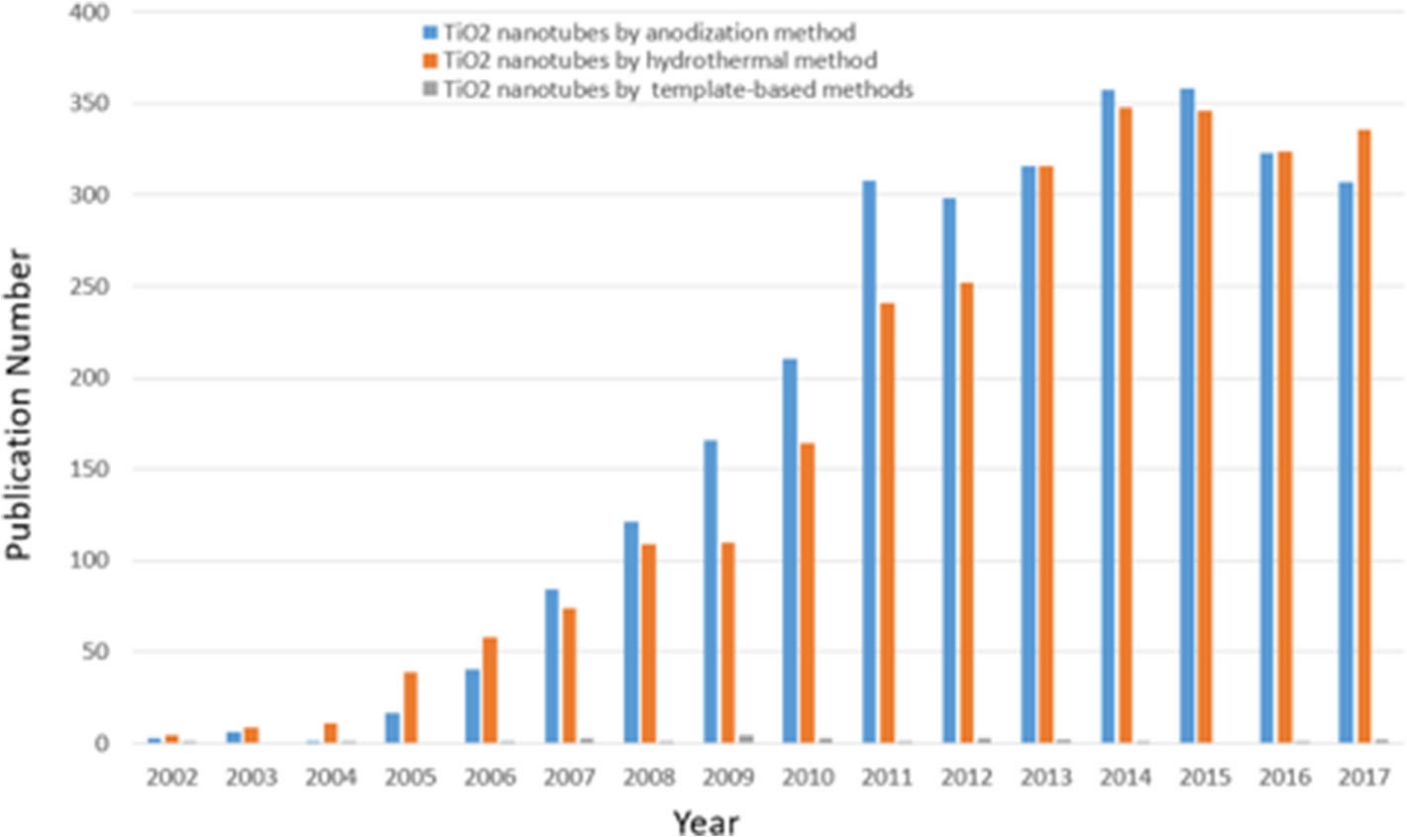
Research trend. The number of papers broken down per year related to TiO_2_ nanotubes differentiated by different synthesis methods from 2002 to 2017. (Data were collected from Science Citation Index Expanded using titania nanotubes, and anodization or hydrothermal methods or template-based methods as keywords)

Although the most explored nanomaterial so far is still the carbon, another class of nanotubular materials, which are usually based on transition metal oxides, has attracted considerable interests over the past 20 years. The first effort to form anodized titania nanotubes was made by Assefpour-Dezfuly [13] who used alkaline peroxide treatment followed by electrochemical anodization in an electrolyte containing chromic acid. And since Zwilling et al. reported that they produced the first self-organized nanotube layers on Ti substrate by electrochemical anodization in chromic acid electrolytes containing fluorine ions in 1999, the field has expanded enormously quickly [14]. Over the past decade, more than 33,800 papers with a keyword of “titania nanotubes” have been published. Figure 1 gives the total publication broken down per year in the field of TiO_2_ nanotubes and makes a comparison among different synthetic methods in the period 2002–2017 which not just shows an exponential growth trend but apparently indicates that the self-organized anodic TiO_2_ nanotube arrays get much attention with great potential and advantages. Lately, Lee et al. has given a comprehensive and up to date view in the field of anodic titania nanotubes which almost covered all aspects including growth, modifications, properties, and applications with a brief of different synthesis approaches [15]. Compared with other preparation methods like hydro/solvothermal [16–18] and template-assisted methods [19, 20], direct oxidation turns out to be a simple technique with strong operability in which way the desired controllable nanostructure via adjusting size, shape, and the degree of order can be grown by means of optimizing the oxidation parameters such as the applied potential, time, temperature, pH, and the composition of the electrolyte [15]. Owing to the particular geometry, the self-aligned oxide nanotube layers which have highly organized structure and surface-volume ratio are representing unique properties, such as a very high mechanical strength, and the large specific surface area, even providing electronic properties like high electron mobility rate or quantum confinement effects [15, 21]. Furthermore, electrochemical anodization is a low-cost process and not limited to titanium but also can be suitable for other transition metals Hf [22], Zr [23], Nb [24], Ta [25], V [26] or alloys TiAl [27], and TiZr [28]. The present review will still focus on TiO_2_ nanotubes grown by self-organized electrochemical anodization from Ti metal substrate. Besides, we will emphasize the synthesis of this type of self-organized titania nanotube layers and the means to influence the size, shape, the degree of order, and crystallized phases via adjusting the anodization parameters and the subsequent thermal annealing, including four different generations differing from electrolytes species and the defined two-step anodization, etc. The relationship between dimensions and properties of the anodic TiO_2_ nanotube arrays will be presented. The latest progress and significance of the research on formation mechanism of anodic TiO_2_ nanotubes are briefly discussed. We will show the most promising applications reported recently in biomedical directions and modifications carried out by doping, surface modification, and thermal annealing toward improving the properties of anodically formed TiO_2_ nanotubes. We also consider unsolved issues and possible future directions of this field. The main paragraph text follows directly on here.

## Synthesis of TiO_2_ Nanotube Arrays by Electrochemical Anodization

In recent years, while many various forms of nanostructured titanium dioxide including nanorods, nanoparticles, nanowires, and nanotubes have been successfully developed [29–31], nanotubes have attracted increasing interests for technological applications due to the unique self-assembled structure with a large interfacial area and convenient controlling of the size and shape, which can be applied to surface area-dependent applications as a better candidate. A number of excellent reviews [1, 2, 15, 32–34] are available for dealing with the features of TiO_2_ nanomaterials categorized with different synthetic methods. The electrochemical anodization is proved to be one of the most effective methods to obtain the titania nanotubes as a relatively simple technology that can be automated easily. We will specify the main techniques to fabricate anodic TiO_2_ nanotubes below.

### Self-organized Anodic TiO_2_ Nanotube Arrays

**Fig. 2:**
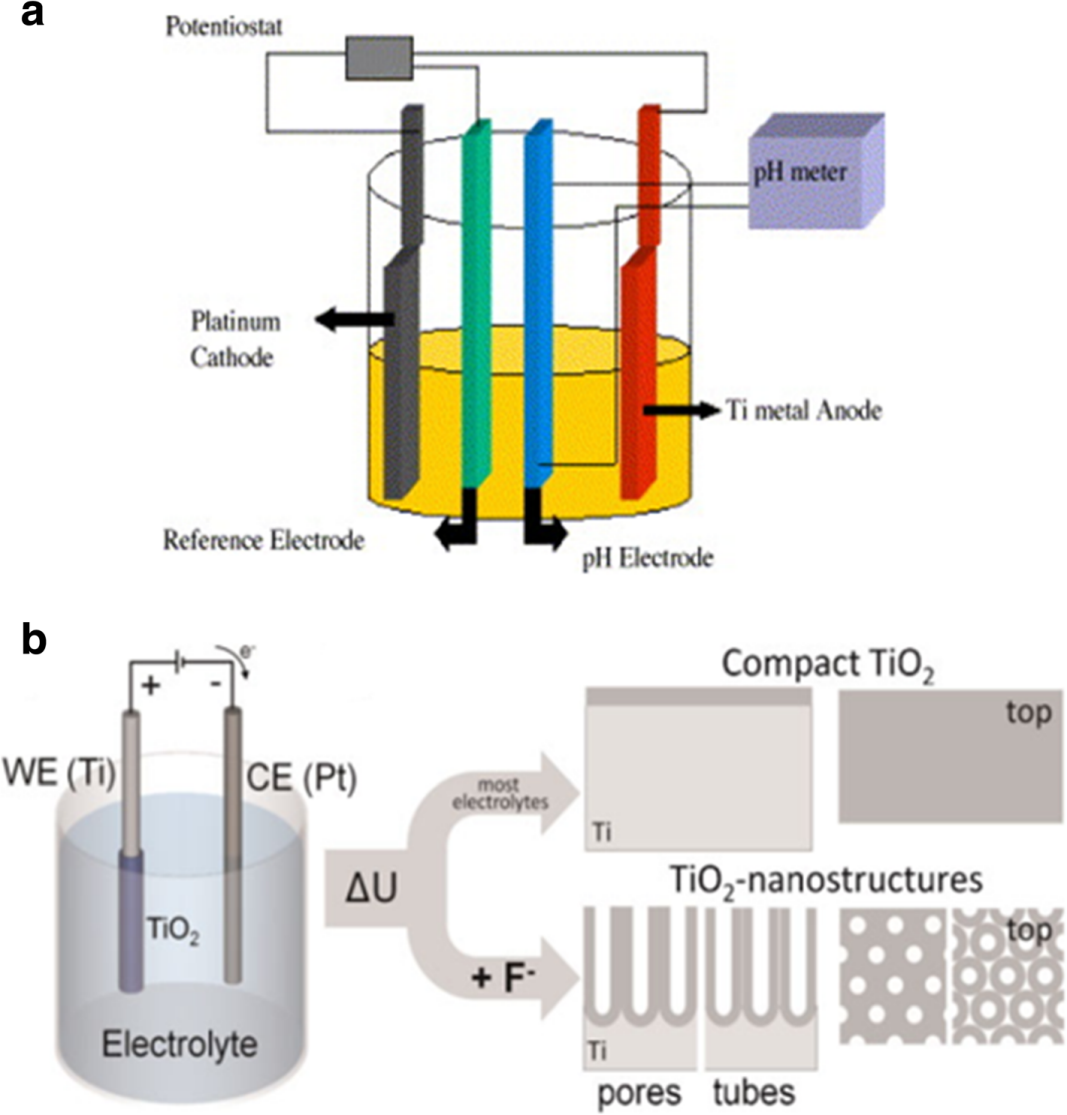
Schematic set up. **a** Illustrative drawing of a three-electrode system with the prepared Ti foil as the working electrodes, platinum as the counter electrode, and usually Ag/AgCl as a reference electrode, while the pH electrode as a pH meter. Reproduced from ref. [35]. **b** Illustrative drawing of a simple two-electrode system composed of Ti foil as anode and inert metal electrode as cathode. Anodization leads to different anodized oxide layer under different conditions. In most neutral and acidic electrolytes, a compact titania can be formed. But if dilute fluoride electrolytes are used, nanotubular/nanoporous oxide layers will be directly attached to the metal surface. Reproduced from ref. [36]

**Fig. 3:**
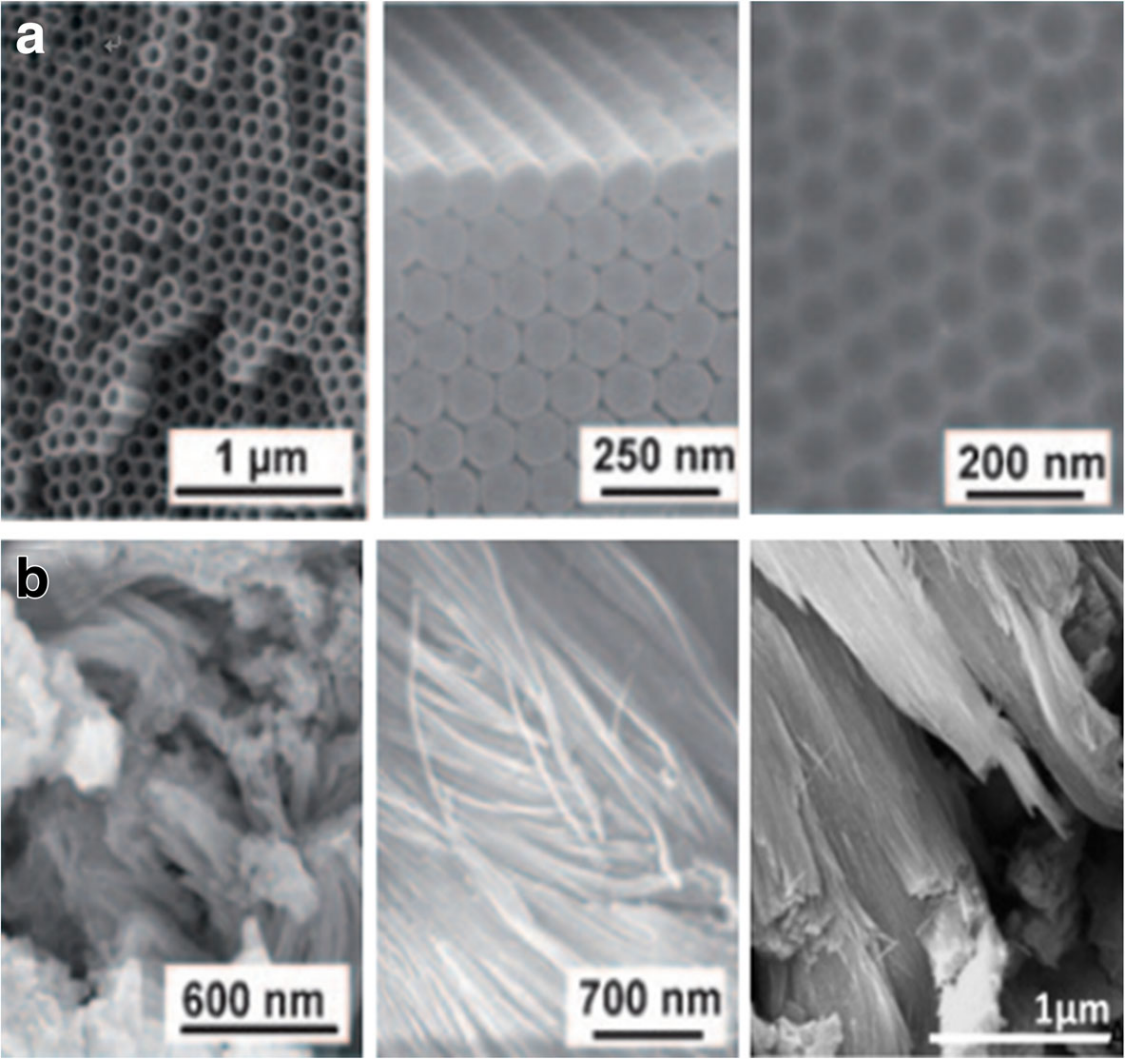
SEM images of anodized TiO_2_ nanotube layers by different anodization processes of Ti. **a** The highly ordered TiO_2_ nanotubes (in top and side view) are obtained in organic electrolyte systems, with self-ordered surface dimples (right) which in fact are metallic surfaces when the tube layers are removed. Reproduced from ref. [1]. **b** The disordered TiO_2_ nanotubes are grown in patches on the surface area and fused together to bundles in chloride containing electrolyte by an ultrafast anodization technique known as rapid-breakdown anodization (RBA). Reproduced from ref. [1] and [37]

**Fig. 4:**
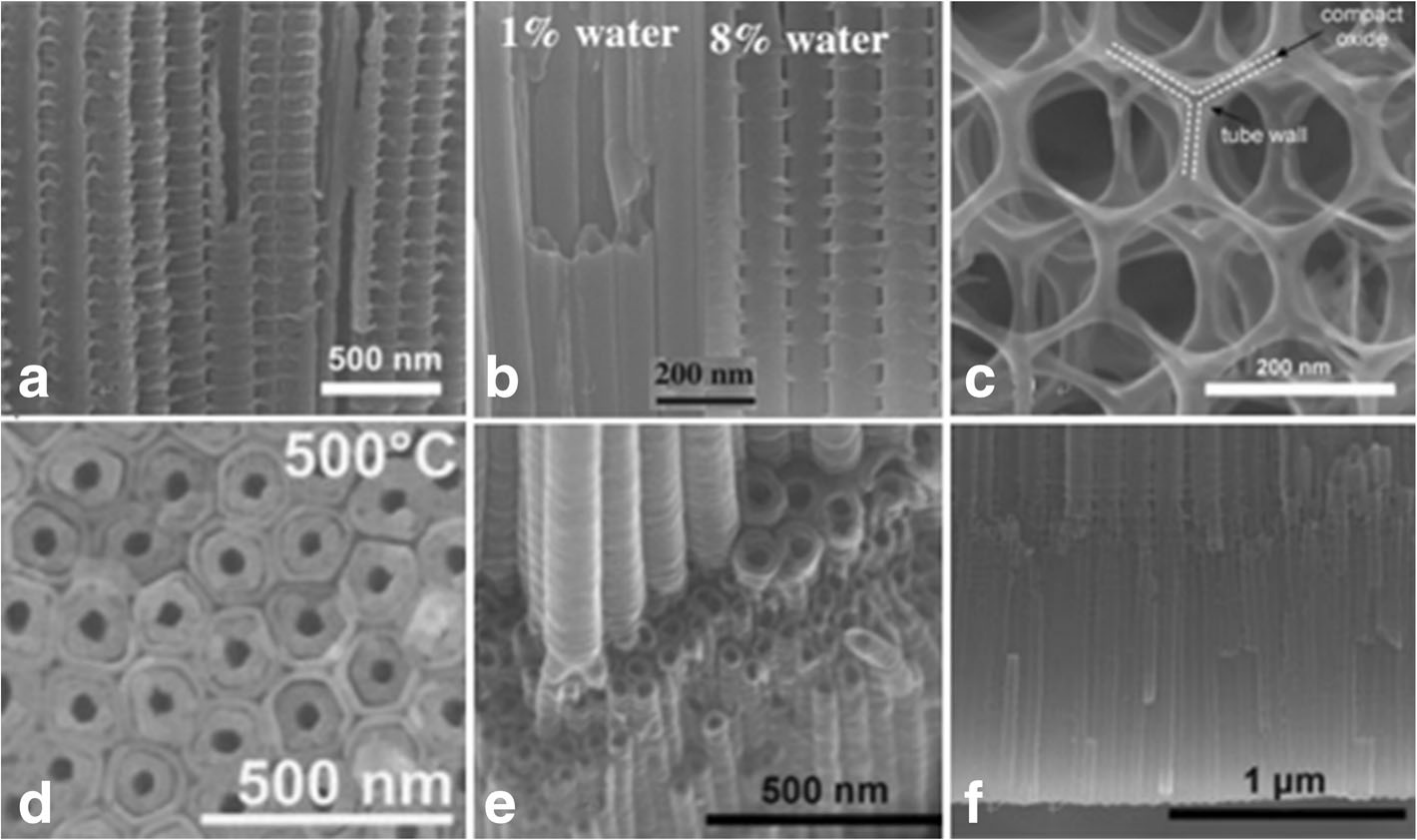
SEM images of advanced TiO_2_ nanotube morphologies. **a** Bamboo-type reinforced TiO_2_ nanotubes are fabricated under specific alternating-voltage (AV) conditions in ethylene glycol consisting of 0.2 mol/L HF, with a sequence of 1 min at 120 V and 5 min at 40 V. Reproduced from ref. [38]. **b** Transition from smooth to bamboo-like TiO_2_ nanotubes can be induced by anodization with controlled water addition (water contents:1 to 8%) to a 0.135 M NH_4_F/ethylene glycol electrolyte reproduced from ref. [39]. **c** The 2D nanolace structures are obtained under voltage cycling carried out for an extended period of time in the fluoride containing electrolyte, with a sequence of 50 s at 120 V and 600 s at 0 V. Reproduced from ref. [38]. **d** The double-walled TiO_2_ nanotubes are grown by anodization of Ti in a fluoride containing ethylene glycol electrolyte at 120 V after annealing at 500 °C with a heating rate of 1 °C s^−1^. Reproduced from ref. [40]. **e** The branched nanotubes can be observed by voltage stepping, first at 120 V (6 h) and then at 40 V (2 h). Reproduced from ref. [38]. **f** The double-layer nanotubes with equal or two different tube diameters can be seen. Reproduced from ref. [38]

As extensively studied, the titania nanotube layers can be formed under a specific set environmental conditions. The oxidation device consists of three parts: (I) a three-electrode system with the prepared Ti foil as the working electrode which is degreased by sequentially sonicating in acetone, ethanol, and deionized water, platinum as the counter electrode and usually Ag/AgCl as a reference electrode (Fig. 2a), while the pH electrode sometimes is added to obtain the ultimate concentration of F^−^ and HF [35] or another simple two-electrode system composed of Ti foil as anode and inert metal electrode as cathode (Fig. 2b) [36]; (II) generally, fluoride ion, chloride ion, chromium ion, bromide ion, or perchlorate containing electrolytes; and (III) a DC power supply. There are two main features influenced by the anodization conditions of formation affecting the promising applications of titania nanotubes: (I) geometry: size, shape, the degree of order, crystallized phases, etc. and (II) properties in chemical, physical, and biomedical. In other words, via controlling the electrochemical anodization parameters (applied potential, the duration of anodization, electrolyte system including the concentration of the fluorine ions, and water in the electrolyte, electrolyte temperature, electrolyte pH, etc. which will be discussed in more details in the “Synthesis of TiO2 Nanotube Arrays by Electrochemical Anodization” section), one can fabricate different titania nanostructures such as a flat compact oxide [1], a porous layer [1, 36], disordered TiO_2_ nanotube layers growing in bundles [37], or finally a highly organized regular TiO_2_ nanotubes or advanced nanotubular layer: branched tube [38], bamboo-like [38, 39], double-walled [40], nanolace [38], or double-layer [39] structures in which way properties could be found differently. Figures 3 and 4 display field-emission scanning electron microscopy (FE-SEM) images of the typical examples of such TiO_2_ nanotube morphologies.

(At present, TiO_2_ nanotube arrays with tube diameters ranging from 10 to 500 nm, thickness of layers ranging from a few hundred nanometers to 1000 μm, and wall thickness ranging from 2 to 80 nm can be obtained [15, 41].)

**Fig. 5:**
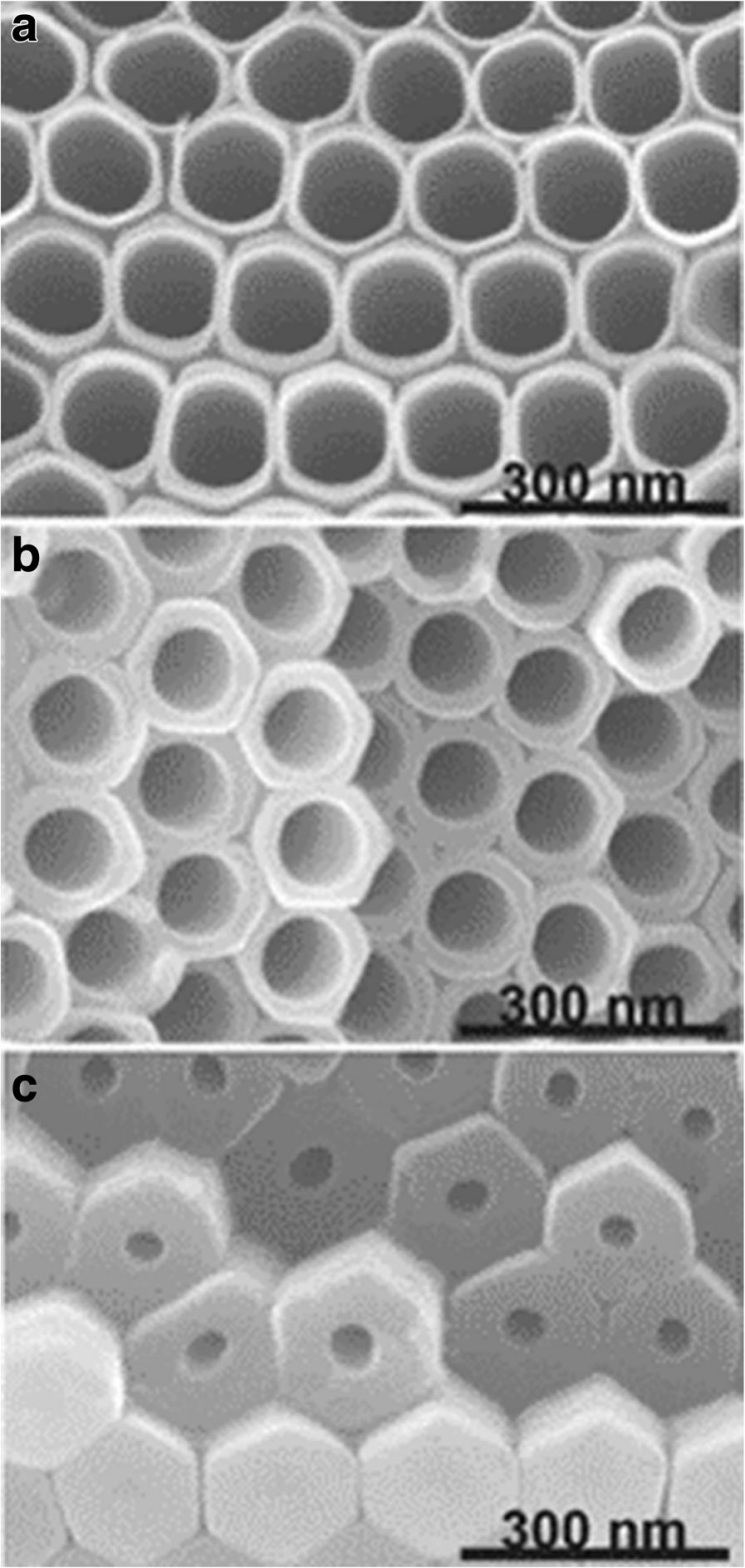
SEM images of TiO_2_ nanotubes. The nanotubes are formed in ethylene glycol electrolytes containing 0.27 M NH_4_F by repeated anodization of Ti. The cross sections are taken at the top of the layer, in the middle, and at the bottom of the layer. Reproduced from ref. [43]

It was two decades ago when Masuda and Fukuda for the first time reported the highly ordered porous alumina through adjusting the anodization conditions to an optimum [42]. Later on, researchers spent their efforts to make similarly organized structures also for TiO_2_ nanotube layers. And there are three crucial factors affecting the degree of order in anodic TiO_2_ nanotube arrays (in accordance with polygons in the layers and the standard deviation in tube diameter): the Ti substrate, the applied voltage, and the repetitive anodization [33, 43]. It is obvious that fewer flaws in the arrangement can be obtained for high purity material at the highest possible voltage below dielectric breakdown [33] and the ideally hexagonal self-ordered TiO_2_ nanotubes as shown in Fig. 5 can be improved significantly by secondary tubes growth [43]. Sopha et al. showed impurities strongly influence the resulting different dimensions and ordering of nanotubes after the second anodization [44]. Moreover, the crystallographic orientations of the Ti substrate grains have been revealed to be crucial effects in growth characteristics of TiO_2_ nanotube arrays by electron backscatter diffraction (EBSD). Leonardi et al. found that nanotubes can only be observed with an orientation that enables a valve metal oxide to form on grains allowing penetration of fluoride ions through the oxidation film where 1 M (NH_4_)H_2_PO_3_+0.5 wt% NH_4_F were used as electrolyte [45]. Similarly, Macak and co-workers reported that no nanotube growth on grains is retarded in the widely used ethylene glycol-based electrolyte compared to the case of using aqueous electrolyte, as known from the last literature [46]. On the polished Ti sheet, grains with [0 0 0 1] orientation or close to this turned out to be the ideal grains and utilizing single-crystalline Ti with ideal orientation would be a great advance to obtain the most uniform nanotube arrays [46].

Nevertheless, there are still some defects influencing the degree of order. Lately, it has been further extended by uniform nanoimprinting Ti. Kondo et al. figured out a throughput fabrication of an ideally ordered anodic TiO_2_ by nanoimprinting Ti surface or a two-layered specimen with an Al layer on the top and the Ti layer at the bottom using a Ni mold with ordered convexes. And the TiO_2_ layers could be generated in a more orderly way where the shallow concaves of the pre-textured pattern acted as initiation sites by the subsequent anodization in NH_4_F ethylene glycol solution [47, 48]. Following closely, Sopha et al. firstly covered a TiN-protecting layer on Ti substrate prepared by atomic layer deposition (ALD) before the pre-texturing carried out by focused ion beam (FIB) and the subsequent anodization using ethylene glycol electrolyte to produce perfectly hexagonally arranged nanotube layers with a thickness of 2 μm, which can restrict the nanotubes only to grow on the given initiation sites and extend anodization time without any defects [49].

### Formation Mechanism of Anodic TiO_2_ Nanotubes

Anodic oxidation technology and researches on formation mechanism of anodic TiO_2_ nanotubes has captured wide attention for a long time from a broad diversity of disciplines. The mechanism research Diggle reported in 1969 about the films of compact anodic oxide and porous anodic oxide [50] now still plays an extremely important guiding role. A significant amount of recent works show that transition from pores to tubes is of a gradual nature [1, 27, 36]; however, the fully theoretical model and reasoning were not given.

**Fig. 6:**
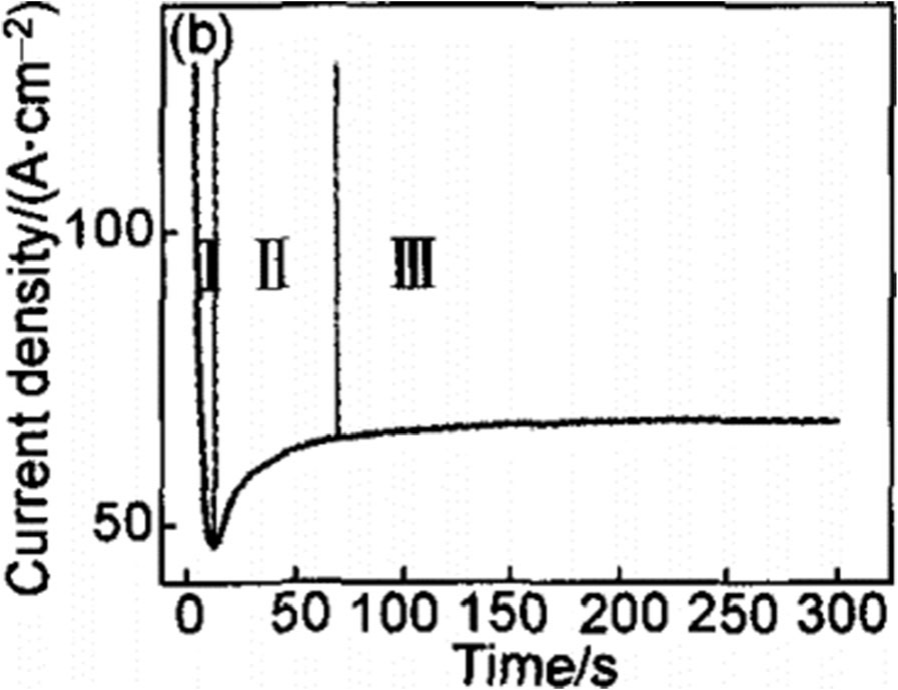
Typical current time curve under a constant voltage in electrolytes containing fluoride. The transient can be divided into three distinct regions (**I**–**III**). (**I**) In the first part, there is a sharp current decay. (**II**) In the second part, the current begins to rise again with a time lag. (**III**) In the third part, the current reaches a steady state reproduced from ref. [51]

**Fig. 7:**
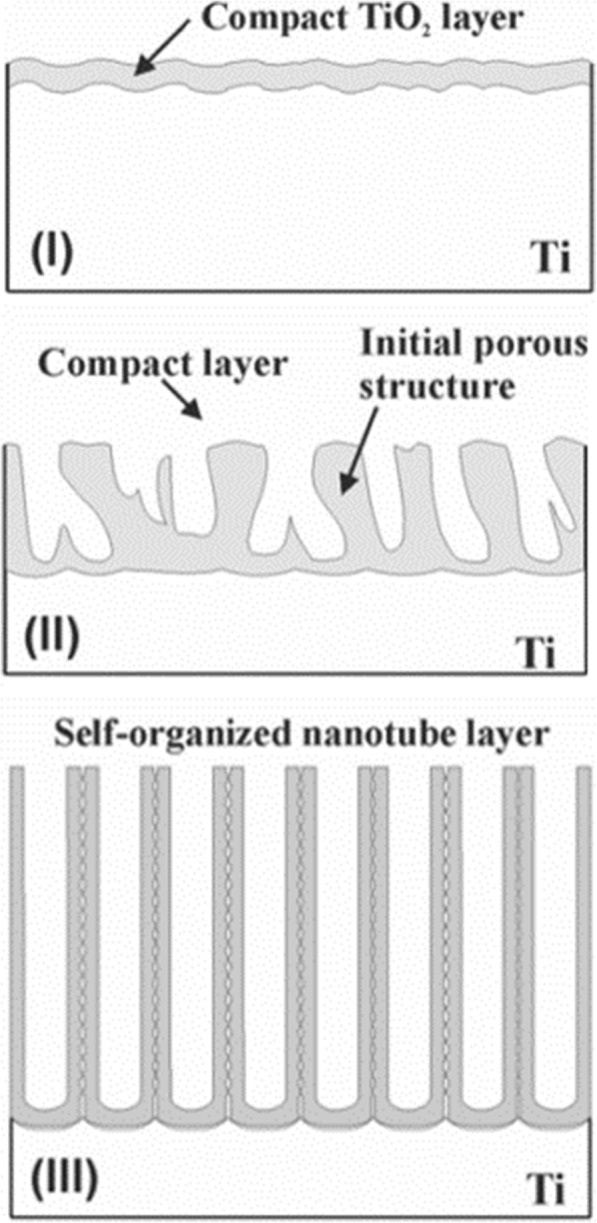
The forming process of TiO_2_ nanotube arrays. The formation of TiO_2_ nanotube arrays can be divided into three different morphological stages (**I**–**III**). (**I**) A barrier oxide is formed. (**II**) The surface is locally activated and pores start to grow randomly. (**III**) Self-organized nanotube layer is formed reproduced from ref. [33]

Conventional field-assisted dissolution (FAD) is the most acceptable theory [1, 33, 51]. That is in the process of electrochemical anodization, TiO_2_ nanotube arrays are formed by self-organization of titania because of three relatively independent procedures: electrochemical oxidation of Ti into TiO_2_, the electrical field-induced dissolution of TiO_2_, and the fluorine ion-induced chemical dissolution of TiO_2_, reaching a delicate balance. As a characteristic current time curve shown in Fig. 6 for electrolytes containing fluoride that lead to nanotube formation [51] and a typical image that can help to illustrate the formation process schematically [33] shown in Fig. 7, the transient can be divided into three distinct stages: (I) In the first part, there is a current decay, caused by a newly formed barrier oxide, when the two major processes, the inward migration of O^2−^ ions toward the metal/oxide interface and the outward migration of Ti^4+^ ions toward the oxide/electrolyte interface, achieve a balance. (II) In the second part, the current begins to rise again with a time lag caused by the increasing surface area of the anode. The shorter the lag is, the higher the fluoride concentration will be due to the fluoride-induced dissolution of the formed TiO_2_, and pores start to fabricate randomly which subsequently turn out to be the initial formation of TiO_2_ nanotubes. (III) Then, the current reaches a steady state, when the pore growth rate at the metal oxide interface and induced dissolution rate of the formed TiO_2_ at the outer interface reach an equilibrium situation. Thus, the final tubes become increasingly v-shaped, that is, the tops of the tubes possess significantly thinner walls than their bottoms where the tubes are closed-packed. The gradient in the tube wall thickness in Fig. 5 can be ascribed to different exposure time and concentration to the electrolytes along the tubes [43].

However, this theory cannot explain the phenomenon of separation into tubes, as opposed to a nanoporous structure clearly yet, and Fahim et al. observed that under appropriate voltages it is possible to obtain titania nanotubes in sulfuric acid solution without fluoride ions, in which case, the I-t curve resembled the one we just discussed above [52]. As Houser and Hebert pointed out, growth mechanism have not yet been developed to explain the quantitative relationships between the process of titania porous membrane and I-t curve [53]. Because the interpretation is not convincing enough, new points about the mechanism appear recently such as viscous flow model and growth model of two currents. With regard to these mechanisms, a review [51] shows lots of limitations for the traditional field-assisted dissolution theory and makes some explanations on latest progress and significance of the research on viscous flow model and growth model of two currents.

### The Effect of Anodization Conditions Affecting Geometry and Properties

The composition and concentration of the electrolytes have significant influence in the nanotube arrays formation. According to the difference of electrolytes we use, the development is basically divided into three stages: Table 1 summarizes the anodization conditions and dimensions of the resulting TiO_2_ nanotube arrays in the three generations investigated by different research groups to date.

**Table 1:** TiO_2_ nanotubes arrays fabricated through anodic oxidation in the three generations: electrolyte compositions, anodization conditions, and size of the resulting nanotubes

Generation	Electrolyte	Condition	Diameter	Thickness of layers	Ref
First generation	0.5 wt% HF in aqueous solution	20 V 20 min	60 nm	250 nm	[54]
second generation	0.1MKF+1MH_2_SO_4_+0.2Mcitric acid	25 V 20 h	115 nm (inner)	4.4 μm	[57]
	1MNa_2_SO_4_+0.5wt%NaF	20 V 6 h	100 nm	2.4 μm	[58]
Third generation	0.5 wt% NH_4_F	20 V	40 nm	7 μm	[59]
	In glycerol	13 h			
	0.3 wt% NH_4_F	60 V	100 nm	45 μm	[63]
	In ethylene glycol	18 h			
	0.25 wt% NH_4_F	60 V	160 nm	134 μm	[60]
	In ethylene glycol	17 h			

The first generation: hydrofluoric acid (HF)-based aqueous electrolytes

The milestone is that Gong et al. for the first time presented the uniform titania nanotube arrays by anodic oxidation of Ti in HF-based aqueous electrolytes [54]. In HF aqueous solution electrolytes, where pH is relatively low meaning high concentration of hydrogen ions, the chemical dissolution of TiO_2_ induced by fluorine ions plays the dominant status in the anodization process [55]. A dynamic equilibrium was achieved in a short period of time in the process of forming titania nanotubes, and therefore, the maximum achievable nanotubes length was restricted to approximately 0.5 μm [54–56].

The second generation: buffered electrolytes

In the subsequent work, in order to reduce chemical dissolution lengthening the tubes, Cai et al. demonstrated that by adding weaker acids such as KF or NaF into a buffered solution and adjusting the pH to weakly acidic (pH = 4.5) with sulfuric acid or sodium hydroxide, nanotubes approximately 4.4 μm in length were achieved [57]. PH value affects the hydrolyzation of the titanium ions which turn out to interfere the electrochemical etching and chemical dissolution. Cai et al. also pointed out that lower pH values produce shorter but clean nanotubes and higher pH values result in longer nanotubes but unwanted debris [57]. As the pH value goes up, the rate of hydrolysis will increase, in turn slowing down the chemical dissolution, leading to longer nanotubes while alkaline solution is not suitable for nanotubes growth [57, 58]. It is demonstrated that in neutral NaF electrolyte at proper voltage much longer nanotubes could be obtained than in acidic solutions by Macak et al. [58]. Given a particular voltage in fluoride containing electrolyte, by adjusting the pH gradient, the required aspect ratios and thickness of layers could be achieved [59].

The third generation: polar organic electrolytes

Electrolytes such as glycerol [59], dimethyl sulfoxide [60], formamide or diethylene glycol [61, 62], ethylene glycol [41, 63], containing fluoride species such as NH_4_F, NaF, and KF gradually appear. Macak and co-workers took the lead in using viscous glycerol electrolyte to fabricate titania nanotube arrays with the thickness of approximately 7 μm and an average tube diameter of 40 nm [59]. It is demonstrated that higher aspect ratio TiO_2_ nanotubes can be grown in such polar organic electrolyte due to proper control of electrolyte pH reducing chemical dissolution of the titania [64]. Paulose et al. formed nanotubes approximately 134 μm in length, prepared using ethylene glycol containing 0.25 wt% NH_4_F at an anodization potential of 60 V for 17 h [60]. Soon afterwards, more than 250-μm-thick TiO_2_ nanotube arrays were reported by Albu [65]. Besides, the water content plays a dual role in the process: it is indispensable for the formation of titania, but it also speeds up the chemical dissolution [63]. Hence, how to shrink the effect of water content to a minimal significance is required for increasing the thickness and degree of order of the TiO_2_ nanotube arrays. In general, limiting water content to less than 5% is the key to achieve very long nanotubes successfully [60], and a minimum amount of water content (0.18 wt%) is required to form well-organized titania nanotubes [66]. It was reported that with the addition of water, the current density recorded decreased which was the highest in anhydrous ethylene glycol solution [66]. Paulose et al. first reported formation of self-organized hexagonally titania nanotube arrays with approximately 1000 μm in length at 60 V for 216 h in ethylene glycol containing 0.6 wt% NH_4_F and 3.5% water [41]. Another noticeable phenomenon is that smooth tube walls are grown at low water content, while ripples on side walls are formed at higher content as shown in Fig. 4b [59, 67]. As by far the most employed type of electrolytes, ethylene glycol containing water and fluoride ions always leads to a double-walled nanotube structures (Fig. 4d) [40, 68–70], while the inner layer can be removed by a suitable annealing treatment followed by a simple chemical etching process. After removal of the inner shell, the widened tubes allow a layer-by-layer decoration with nanoparticles using a repetitive approach based on TiCl_4_-hydrolysis [71]. Whereas single-walled tubes showed significantly enhanced conductivity and electron transport times in dye-sensitized solar cells (DSSCs) [71, 72] where the thickness of the entire tube is basically the same and the inner shell no longer appears, Mirabolghasemi et al. made a comparison between the double- and single-walled tubes and presented desired single-walled tubes with an addition of dimethyl sulfoxide (DMSO) into electrolytes with 1.5 M H_2_O and 0.1 M NH_4_F [72].

Recently, non-fluoride-based electrolytes have been reported to grow TiO_2_ nanotube arrays which may be considered as the fourth synthesis generation including hydrochloric acid, hydrogen peroxide, perchloric acid solutions, and their mixtures [73, 74]. Allama and Grimes described well-developed nanotube arrays with 300 nm in length, 15 nm in inner diameter, and 25 nm in outer diameter were obtained in a 3 M hydrochloric acid (HCl) aqueous electrolyte at oxidation voltages between 10 and 13 V. But adding a low concentration of H_3_PO_4_ resulted to a change from nanotubes to rods. They further suggested that they were unable to achieve self-organized nanotube arrays in HCl-containing electrolytes at the concentration of lower or higher than 3 M [73]. Allama found out that adding hydrogen peroxide to the hydrochloric acid containing aqueous solution could be a possible method to lengthen the titania nanotubes which possesses a strong oxidizing property following a thicker oxide layer, demonstrating that fluoride ions can successfully be replaced by chloride ions in the growth of nanotube arrays [74]. Besides, ionic liquids without addition of free fluoride species have been treated as another type of solvent system for titania nanotubes in recent years [75, 76].

In addition to the standard parameters, the geometry of the resulting nanotubes is dependent on the repetitive use of electrolyte (the “used solution effect”). In comparison with the tubes obtained with fresh solutions, using once-used solutions, exhibited an increase in nanotube length and a better quality where the nanotube growth rate achieved is consistently higher for once-used solutions at 60 V and above [77] and a slightly different but distinguishable current transient behavior could be noted [66]. Moreover, no nanotubular structure but an oxide film was obtained in the twice-used solution because of the depletion of F^−^ species [78]. However, Sopha et al. investigated different ages of ethylene glycol based electrolytes on the morphology of TiO_2_ nanotubes showing that in older electrolytes the arrays exhibit lower aspect ratios [79].

#### Applied Potential

Anodization voltage is the critical factor controlling tube diameters [80, 81]. The dimension of the nanotube arrays can be predicted just simply by applying the suitable range of voltage called potential window across the electrode [67]. At a low voltage, less electric field dissolution occurs, forming TiO_2_ nanotubes with smaller diameters. If the voltage is too low, the TiO_2_ layer becomes compact but no nanotubular structure can be observed. On the contrary, a spongy-like porous structure will be seen when the voltage is too high. With controllable voltage, the diameter of the nanotubes is proportional to the voltage [81]. Furthermore, studies show that the range of voltage forming nanotubes is also related to the electrolyte system. In aqueous electrolytes, the potential window should be controlled from 10 to 25 V, which in organic electrolytes is much wider between several volts and some hundred volts. Wang and Lin found out the fact that in aqueous electrolytes, the anodization potential exhibits significant influence on the growth of TiO_2_ nanotube arrays, which exhibited slight influence in non-aqueous electrolytes in this regard [82]. The voltage dependence has a significant reduction in non-aqueous electrolytes which is attributed to a large extent to the low conductivity of organic electrolytes [83, 84].

#### The Duration on Anodization

The duration of anodization affects the nanotubes mainly in two aspects: (I) the formation of the tubes or not and (II) the length of the tubes. That is, in the early stage of the anodization, a compact TiO_2_ film is formed. If the duration is too short to reach an equilibrium in reaction, the regular nanotube array cannot be achieved instead of a disordered porous layer [67]. With increasing the anodization time, porous structure gradually grows deeper and converts into the TiO_2_ nanotubular array [1, 33, 51]. If other electrochemical parameters are kept unchanged, increase in the nanotube length is observed over time while no significant effect on diameter and tube wall thickness until a steady-state situation occurs [67, 85, 86]. However, due to the decrease of the F^−^ concentration in the electrolyte, where the ion transport rate decreased, the growth rate of nanotubes is reduced. After reaching a stable condition between tube growth at the bottom and chemical/electrochemical dissolution at the top, we will find no further increase in length of the nanotubes [87]. As time continues to go, pipe orifice becomes an irregular polygon resulting in TiO_2_ spikes and coverings which can be seen on the surface of the TiO_2_ nanotube arrays [36]. It is worth mentioning that enlightened by the success of aluminum-repeated anodization for self-organized porous alumina [88], the two-step anodization of titanium for such a highly ordered hexagonally packed nanostructure of titania has appeared [43, 77, 89–91]. After the first-step anodization, the first nanotube layer from the Ti foil should be removed ultrasonically or by using an adhesion tape which leads to a surface where the remaining Ti is covered by comparably ordered dimples. Researches have shown that the former treatment helps to avoid potential mechanical damage to the Ti surface and also improve the structural uniformity of the TiO_2_ nanotubes to a great extent [77, 90, 91]. In the second anodization step, the pretreated Ti foil would be used as anode again with or without changes in parameters of oxidation conditions. It is subsequently found that the highly ordered and vertically oriented titania nanotubes, have greater potential in such fields as photocatalysis [77], photoelectriochemical activity [92, 93], and biological interaction with cells [94] than the disordered nanotubular titania.

#### Electrolyte Temperature

Temperature restricts the growth and quality of titania nanotube arrays, directly affecting the rate of oxide growth, length, and wall thickness of the structure [64, 95]. Wang and Lin first reported the effect of electrolyte temperature in both aqueous and non-aqueous electrolyte on anodic oxidation of titanium [82]. In aqueous electrolyte, with the temperature increasing, a slight diminish in the internal diameters was observed while the external diameters remained the same [68]. The reason may be the dissolution induced by electrical field and fluoride ions are similar while the oxide formation rate is higher than that at lower temperature. In non-aqueous electrolyte containing fluoride ions, the outer nanotube diameter was found to be largely increased by the increasing electrolyte temperature [82]. This may be because at lower temperature, the ion mobility of fluorine in some viscous electrolyte is further inhibited, leading to much slower dissolution of newly formed titania, which subsequently lead to a smaller nanotube diameter. As chemical dissolution rate increases, surface of TiO_2_ nanotubes arrays can easily produce excessive corrosion, resulting in lodging nanotubes and agglomeration. Therefore, the appropriate bath temperature for stable TiO_2_ nanotube arrays is at room temperature [82, 95, 96].

## Modification of Nanotubes Properties

Increasing applications of TiO_2_ nanotubes as a novel semiconductor are closely related to its photoelectriochemical (PEC) performance; however, they are sometimes prevented by two fundamental drawbacks: (I) the wide band gap (3.0 eV for the rutile phase and 3.2 eV for the anatase phase) can only absorb ultraviolet light, which accounts for less than 10% of the sunlight [97], resulting in low average utilization ratio of solar energy and (II) the low electrical conductivity cannot efficiently transfer photogenerated carries. At the same time, the photoelectrons and vacancies can be easily recombined, thus making low electron mobility rate or quantum confinement effects [98]. Hence, post-treatment of TiO_2_ nanotubes is the key to improve the performance of its materials and related devices successfully. Considerable researches have been reported on modified methods to reduce the recombination of photogenerated electron-hole pair rate, speed up the electron transfer rate, and enhance the photoelectriochemical activity of TiO_2_ nanotubes. The research of the methods for the improvement of the photoelectriochemical properties of TiO_2_ nanotubes will be reviewed, including thermal annealing, doping, and surface modification. As for promising modification in biomedical fields, we will present in the application section.

### Thermal Annealing

The crystallinity of the nanotube arrays and their conductivity, lifetime of charge carrier, and photoresponse depend mainly on the thermal annealing temperature and atmosphere [99, 100]. The as-prepared TiO_2_ nanotubes above are amorphous in nature but can be annealed to anatase or rutile phase, or mixtures of both phases relying on the specific temperature [1, 3, 40, 92, 100]. It is demonstrated that amorphous nanotube layers grown in a glycerol-based electrolyte containing fluoride ions have low photocurrents and an incident photon-to-electron conversion efficiency (IPCE) below 5% due to lots of structural defects while anatase phase nanotubes exhibit an IPCE value up to 60% thus attracting more interest to applications such as dye-sensitized or perovskite solar cells [93]. As well in mixed water-glycerol electrolyte with F^−^, Das et al. stated their points that if the self-organized TiO_2_ nanotube arrays with thickness about 1 μm were annealed around 300–500 °C, the anatase phase of TiO_2_ as the most preferred crystalline structure could be observed. The single anatase structure of nanotubes with the best photoelectriochemical properties and the lowest resistivity could be fabricated when annealed at 400 °C. At temperature higher than 600 °C, a track of typical rutile appeared and with a further increase in annealing temperature the percentage and quality of the rutile phase increased [92]. It should be noted that in Jaroenworaluc’s work, rutile phase was detected in anodic nanotube layers grown in aqueous NaF/Na_2_SO_4_ with thickness of approximately 1.5 μm at 500 °C heat treatment and became the dominant phase at 600 °C. Whereas at 550 °C, partial nanotubes began to break down [101]. It begins to cause the collapse of the entire nanostructure formed in aqueous NaF/Na_2_SO_4_ with the continuous increase of temperature (800–900 °C) or the extended annealing time [3]. While for extended temperature, the crystalline structure of the nanotubes completely converts to rutile phase at above 900 °C [3]. Some researchers demonstrated a loss of the typical single-walled nanotube layers morphology when the annealed temperature rose above 580 °C [102]. Besides the whole annealing process especially the heating rate controls, the morphological structures of the entire nanotube arrays [40]. The double-walled nanotube layers prepared from ethylene glycol (containing less than 0.2 wt% H_2_O), with the addition of HF and H_2_O_2_, have such a high stability that can keep their structure intact until temperature is higher than 900 °C with a heating rate of 1 °C s^−1^. However, the double-walled nanotubes begin to collapse as soon as the temperature reaches 500 °C when the heating rate is 25 °C s^−1^. Most extraordinarily, with the high speed of 50 °C s^−1^ the entire separated nanotubes fuse into a highly ordered porous membrane [40]. Xiao et al. obtained crystallized titania nanotubes arrays with calcination in different gases like dry nitrogen, air, and argon indicating nanotubes in dry nitrogen appeared to have enhanced electrochemical and photoelectrical properties who also found out that with the increasing temperature internal diameter decreased while wall thickness increased at the expense of nanotubes length [103].

**Fig. 8:**
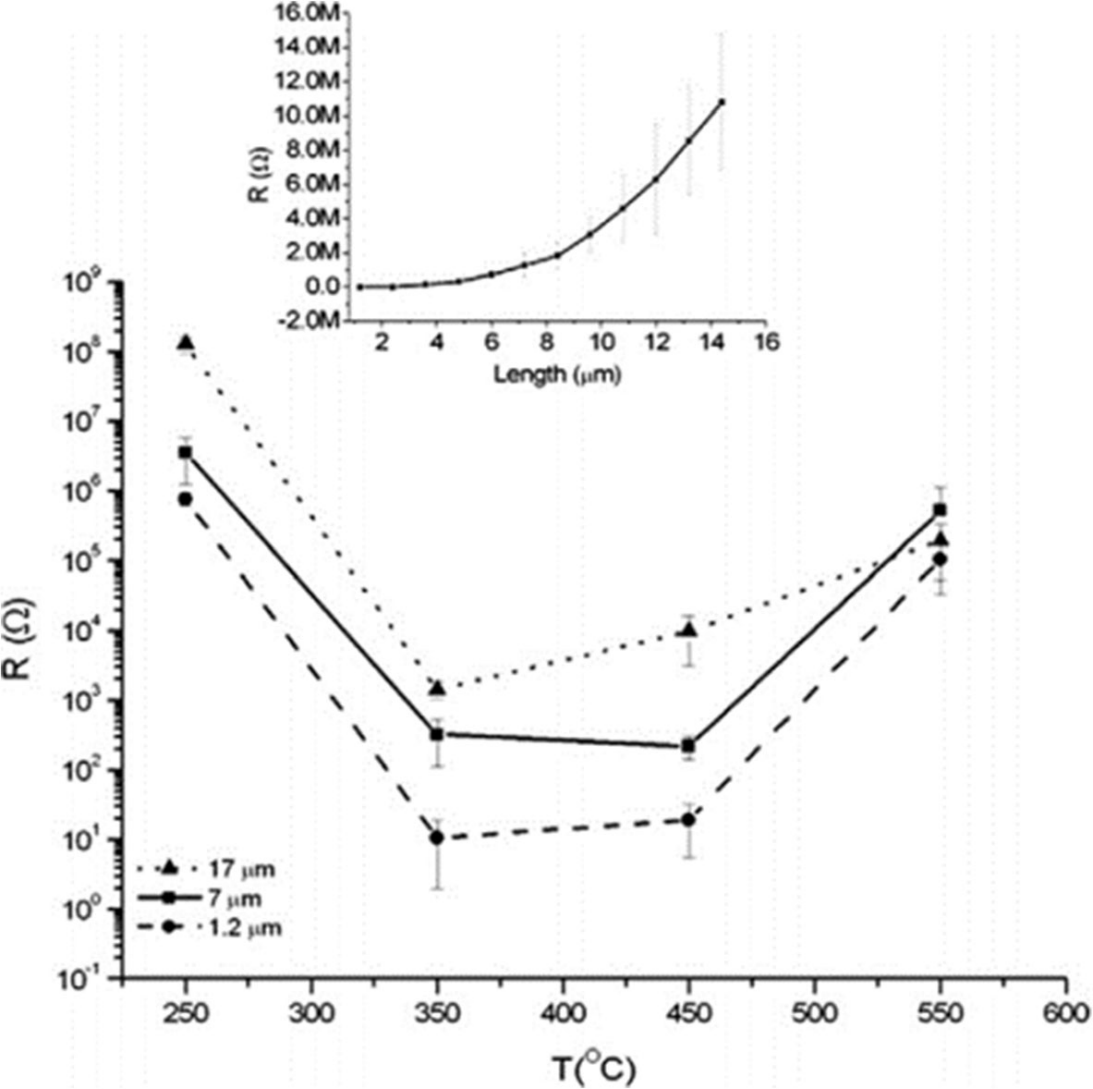
Electrical resistance as a function of the annealing temperature for the different nanotube layer thicknesses. The curve shows electrical resistance measurement for different titania nanotube arrays grown in ethylene glycol based electrolyte containing HF and water at different temperature and the influence of thickness on resistance. The inset shows more details about the relationship between the thickness of the nanotube arrays annealed at 250 °C and their specific resistivity. Reproduced from ref. [99]

**Fig. 9:**
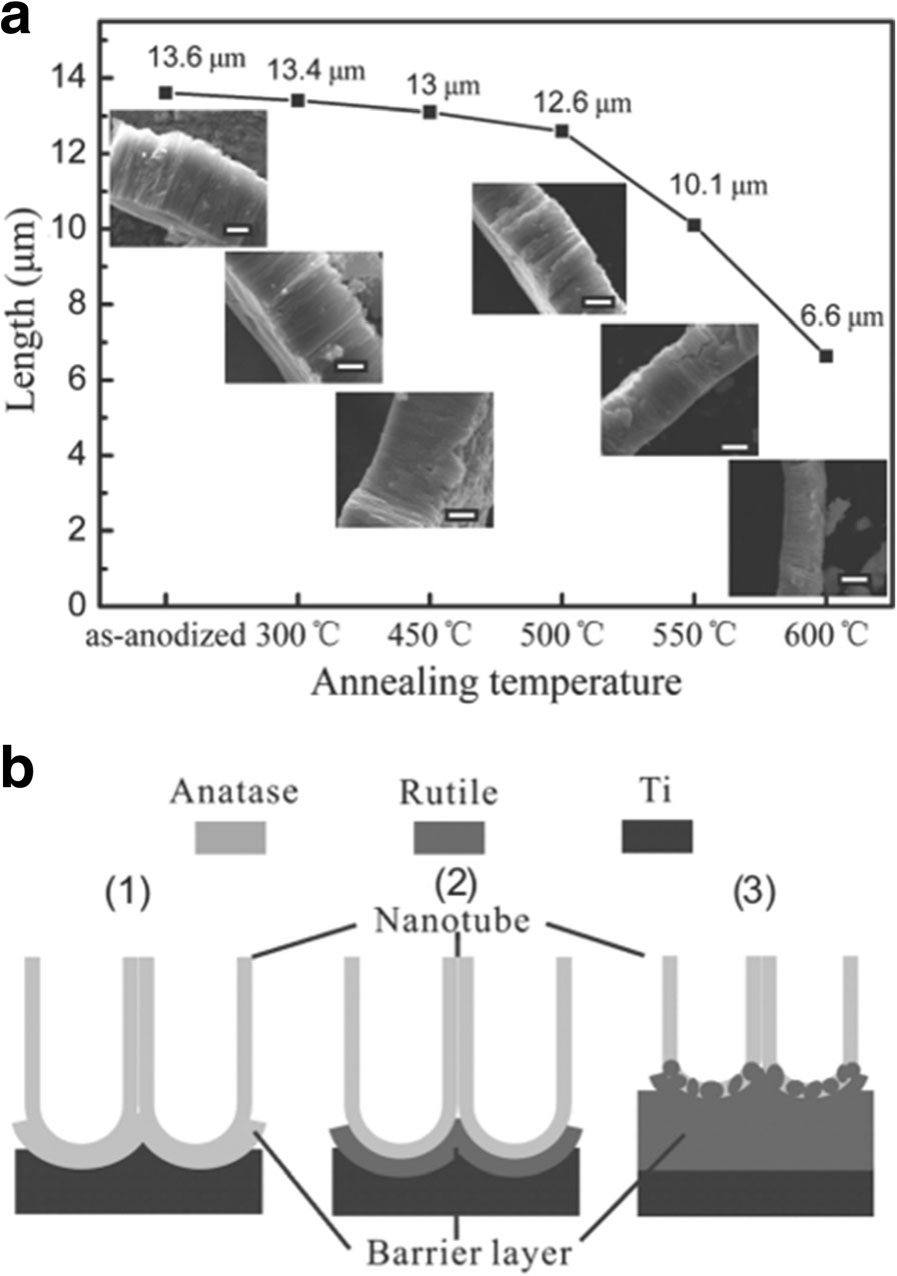
Evolution of titania nanotube arrays at different calcination temperatures. The electrolyte was ethylene glycol containing 0.3 wt% ammonium fluoride and 5 vol% distilled water. **a** The decrease in the thickness of titania nanotube arrays at different annealing temperature from 300 to 600 °C. The insets are corresponding SEM images and the scale bar is 5 μm. **b** The schematic of crystallization process of anodic titania nanotubes annealed at (1) 450 °C, (2) 500 °C, and (3) 600 °C in air. Reproduced from ref. [104]

As shown in Fig. 8, the conductivity along the TiO_2_ nanotubes with three different thickness is strongly affected by annealing temperature. Smallest resistance is observed at about 350–450 °C when the amorphous nanotube arrays are totally converted into anatase layers [99]. And it is evident to see that specific resistivity increases with thicker nanotube arrays which can be shown more clearly in the inset in Fig. 8. Furthermore, calcination temperature is responsible for the decrease in the length of the anatase TiO_2_ nanotubes. As shown in Fig. 9a, increment of temperature between 300 and 500 °C causes the as-prepared nanotube arrays slightly changing in thickness from 13.6 to 12.6 μm. When annealing temperature continuously increases to 600 °C, the average length of the nanotubes decrease dramatically to 6.6 μm. Figure 9b shows conversion from anatase TiO_2_ to rutile phase TiO_2_ occuring at 500 °C when the rutile barrier layer is formed on the bottom of the TiO_2_ nanotube arrays along the anatase nanotubes by consuming the bottom layer if the annealed temperature is further increased. This leads to a length decrease and corresponding photocatalytic activity decline [104].

### Doping

Doping ions or atoms into titania lattice, a substitution within the lattice either at Ti^4+^ or O^2−^ sites, on the one hand, changes the lattice constants and bond energy. On the other hand, it is beneficial to the separation between photogenerated electron and hole pair, which in turn adjusts the band gap and improves the photoelectrochemical performance of nanotubes [15]. The impurity doping has been commonly applied to extend the light absorption onset of TiO_2_ nanotubes by either introducing subbandgap states or adjusting its bandgap width [105]. Lately, co-doping approach has been proposed as a more efficient way to reduce the band gap and adjust energy band level in favor of photoelectriochemical reactions [106, 107]. There are various kinds of doped-elements and preparation methods, and Table 2 summarizes some methods and the doping effects of doped titania nanotubes.

**Table 2:** Some doped-elements, preparation methods, and the doping effects of doped titania nanotubes as based on the classification of metal-doping, non-metal doping, and co-doping

Classification	Raw material	Synthesis	Element	Doping effect	Ref
Metal doping	K_3_Fe(CN)_6_	One-step anodizing 60 V 6 h at 25 °C	Fe	Band gap: 2.85/2.65/2.10/2.03 eV (undoped: 3.18 eV) photocurrent density: 930/1320/675/590 μA cm^−2^ (undoped: < 240 μA cm^− 2^) at 1.5 V under visible light more stable and high photoresponse to visible light	[108]
	Cu(NO_3_)_2_·3H_2_O	One-step anodizing 20 V 1 h at room temperature	Cu	Band gap: 2.65 eV (undoped: 3.20 eV) total amount of H_2_ evolved: 29 μL cm^−2^ 2 h (undoped: 7.6 μL cm^− 2^ 2 h) higher decomposition rate of methylene blue higher stability after multiple reuses	[109]
	K_2_CrO_4_	One-step anodizing 60 V 6 h at 25 °C	Cr	Band gap: 2.82/2.71/2.30 eV (undoped: 3.20 eV) photocurrent density: 360/280/190 μA cm^−2^ (undoped: < 39 μA cm^− 2^) at 1.0 V under visible light total amount of H_2_ evolved: 37/28/12 μL cm^− 2^ 4 h (undoped: ≈ 0 μL cm^−2^) higher stability after multiple reuses	[110]
	Zr(NO_3_)_4_	Two-step anodizing 3/7/10/15 V 1 h	Zr	Higher photocatalytic activities than that of pure TiO_2_ nanotube arrays good photocatalytic stability and could be reused	[111]
	ZnF_2_	One-step anodizing 30 V 15 h at 25 °C	Zn	Band gap: 2.86/2.84 eV (undoped: 3.00 eV) degeneration rate of methylene blue under visible light for 10 h:88/66% (undoped: ≈ 62%)	[112]
	V_2_O_5_	One-pot hydrothermal method at 130 °C 3 h	V	Band gap: 2.91 eV (undoped: 3.18 eV) increased photocurrent density reaction rate (*K*_app_)of rhodamine B: 3 ~ 9 fold as compared to undoped one under UV and visible light	[113]
Non-metal doping	NH_3_	Annealing a flow rate of 400 mL min^−1^ at 500 °C 3 h	N	Band gap: 2.8 eV (undoped: 3.1 eV) Table2: Some doped-elements, preparation methods, and the doping effects of doped titania nanotubes as a based on the classification of metal-doping, non-metal doping, and co-doping. Table 2: Some doped-elements, preparation methods, and the doping effects of doped titania nanotubes as a based on the classification of metal-doping, non-metal doping, and co-doping. Photocurrent density: 1.4 mA cm^−2^ (undoped: 1.6 mA cm^− 2^) at 0.9 V under UV-enhanced PEC activities under visible light and decreased UV light absorption	[105]
	H_2_TiF_6_	Spray pyrolysis	F	Enhancement of surface acidity and creation of oxygen vacancies, increase of active sites	[114]
	H_3_BO_3_	Anodizing 1.8 V 15-60 min	B	Band gap: 2.91 eV (undoped: 3.20 eV), photocurrent density: 311 μA cm^−2^ (undoped: 41.7 μA cm^−2^) at − 0.6 ~ 0.9 V under UV	[115]
	CH_4_	Calcination at 820 °C 18 min in natural gas flame	C	Band gap: 2.84 eV (undoped: 2.92 eV) an additional intragap band: 1.30 eV increased lifetime of photogenerated carriers in the UV	[116]
	K_2_S_2_O_5_	One-step anodizing 20 V 1 h at 25 °C	S	Band gap: 2.61 eV (undoped: 3.20 eV) high stability after multiple reuses photocurrent density: > 1.22 mA cm^−2^ (undoped:< 0.19 mA cm^−2^) at 1.50 V under visible light total amount of H_2_ evolved: 41 μL cm^−2^ 4 h (undoped: ≈ 0 μL cm^−2^)	[117]
	KI、HIO_4_	Two-step anodizing 1.5 V 15 min at 23 ± 1 °C	I	Band gap: 2.95/3.0 eV (undoped: 3.07 eV) enhanced photocurrent density under both visible and UV illumination degeneration rate of methylene blue under visible light for 2 h: 71/65% (undoped: ≈ 31%)	[118]
Co-doping	NH_3_ TaCl_5_	Drop-casting method at 450 °C 30 min	N-Ta	Band gap: 2.5 eV (undoped: 3.1 eV) photocurrent density: 2.5 mA cm^−2^ (undoped: 1.6 mA cm^−2^) at 0.9 V under UV-enhanced PEC activities under both visible and UV illumination	[105]
	NH_3_ (NH_4_)_5_[(NbOF_4_)(NbF_7_)_2_]	Anodizing 45 V at 25 ± 1 °C annealing a flow rate of 100cm^3^ min^−1^ at 550 °C 2 h	N-Nb	Strongly enhanced PEC activities for water splitting under both visible light and UV light	[107]
	K_2_[Ni(CN)_4_]	Anodizing 40 V 2 h at room temperature	Ni-N-C	Band gap: 2.588 ~ 2.972 eV (undoped: 3.062 eV) photocurrent density: 10 times greater than that of undoped one under visible light	[119]

The most typical doped TiO_2_ nanotubes are as follows:

i. Metal-doped TiO_2_ nanotubes such as Nb [107], Fe [108], Cu [109], Cr [110], Zr [111], Zn [112], and V [113]
ii. Non-metal-doped TiO_2_ nanotubes such as N [105], F [114], B [115], C [116], S [117], and I [118]
iii. Co-doped TiO_2_ nanotubes such as N–Ta [105], N–Nb [107], and C–N–Ni [119]

Choiet systematically studied the photoreactivities of 21 metal ion-doped quantum-sized TiO_2_ doping with Fe, Mo, Ru, Os, Re, V, and Rh significantly increases quantum efficiency, while Co and Al doping decreases the photoreactivity [120]. Momeni et al. recently obtained Fe-TiO_2_ nanotube (Fe-TNT) composites using different amounts of irons to decorate anodically formed TiO_2_ nanotubes with potassium ferricyanide as the iron source, indicating that Fe doping efficiently accelerates the photocatalytic performance for water splitting [108]. Not limited to transition metals, other elements including N [105], F [114], B [115], C [116], S [117], and I [118] are successfully explored. Nitrogen-doped TiO_2_ nanotubes turns out to be a promising path to narrow the band gap energy with enhanced photocurrent response in the visible light and the tube length influences the magnitude of conversion efficiency [121, 122]. Kim and co-workers proved that TaOxNy layer-decorated N-TNT (N-doped TiO_2_ nanotubes) as dual modified TNTs have significantly improved both visible (3.6 times) and UV (1.8 times) activities for water splitting [105]. At present, more researches are aimed at co-doping which exhibits remarkable synergistic effect causing a significant improvement on photoelectriochemical properties. Chai et al. grew Gd–La co-doped TiO_2_ nanotubes by an ultrasonic hydrothermal method, enhancing visible light photocatalysts [123]. Cottineau et al. modified titania nanotubes with nitrogen and niobium to achieve co-doped nanotubes with noticeably enhanced photoelectriochemical conversion efficiency in the visible light range [107]. Nevertheless, the mechanism for increasing photoconductivity and synergistic effect of various elements on co-doping remains a further study.

### Surface Modification

Surface modification means decoration on surface of TiO_2_ nanotube arrays with nanoparticles (metal, semiconductors, and organic dyes). Nanowire arrays can also be fabricated by electrodeposition into titanium oxide nanotubes [124]. TiO_2_ nanotube is a semiconductor with a wide band gap, which can only absorb ultraviolet light [97, 125]. Any other nanomaterials which possess a narrow band gap or can absorb the visible light can be used as a sensitizer for titania nanotubes. Silver nanoparticles can be decorated on the tube wall by soaking the titania nanotube arrays in AgNO_3_ solutions and photocatalytically reducing Ag^+^ on a TiO_2_ surface by UV illumination [126]. Ag/TiO_2_ nanotubes show a significantly higher photocatalytic activity and good biological performance compared with neat TiO_2_ nanotubes [126, 127]. Some compositions such as graphene oxide GO [128], CdS [129], CdSe [130], and ZnFe_2_O_4_ [131]. can be modified on TiO_2_ nanotube arrays. Lately, GO have attracted much scientific interest in nanoscale devices and sensors which is easy to combine with nanostructure materials to compose some compounds. Titania nanotubes fabricated by anodization in water-ethylene glycol electrolyte consisting of 0.5 wt% ammonium fluoride (NH_4_F) can be incorporated with GO by cyclic voltammetric method, which achieve higher photocatalytic activity and more effective conversion efficiency (GO-modified vs pure nanotubes = 26.55%:7.3%) of solar cell than unmodified TiO_2_ nanotubes [128]. Semiconductor composite is a method improving the performance of titania nanotubes via, in some specific way, combining two kinds of semiconductors with different band gap [132]. Yang et al. decorated CdSe nanoparticles on the surface of TiO_2_ nanotubes by applying an external electric field to accelerate CdSe nanoparticles in nanochannels resulting in a material with more stable and higher photoresponse to visible light. Furthermore, the degeneration rate of anthracene-9-carbonxylic acid when exposed to the green light irradiation indicating that CdSe dominates the photocatalytic process under visible light [130].

Besides, other oxide nanoparticle deposition such as WO_3_ [133] or TiO_2_ [134] onto TiO_2_ nanotubes by the hydrolysis of a chloride precursor also turns out to augment the surface area and improve the solar cell efficiency. Another very effective approach is to consider organic dyes as sensitizers for TiO_2_ nanotubes to improve its optical properties [135]. Lately, atomic layer deposition (ALD) becomes an established procedure to modify TiO_2_ nanotube layers. ALD appears to be a very uniform and precisely controllable deposition process to functionalize nanotubes in conformably coating the surface of the nanotube layers with one atomic layer after another of a secondary material, such as Pd [136], ZnO [137], Al_2_O_3_ [138], CdS [139], or TiO_2_ [140].

## Biomedical Applications

Historically, the mentioned milestones were reported on the fabrication of titania nanotube arrays contributing to widen the promising applications over the past 20 years in the areas ranging from anticorrosion, self-cleaning coatings, and paints to sensors [141–143], dye-sensitized and solid-state bulk heterojunction solar cells [144–146], photocatalysis [147, 148], eletrocatalysis, and water photoelectrolysis [149, 150]. They also outperform in biomedical directions as biocompatible materials, toward biomedical coatings with enhanced osseointegration, drug delivery systems, and advanced tissue engineering [15, 135, 141, 142, 151]. In the following section, we will give an overview of current efforts toward TiO_2_ nanotubes biomedical applications. Titania nanotubes possess good biocompatibility as they show some antibacterial property, low cytotoxicity, good stability, and cytocompatibility including promoting adhesion, proliferation, and differentiation of osteoblast and mesenchymal stem cells (MSCs) with a high surface area-to-volume ratio and controllable dimensions [152–155].

However, Ti products have inadequate antibacterial ability and efforts have been made to improve their antibacterial properties such as modifications on titania nanotubes for biomedical applications like bioimplant [126, 156].

### Biological Coatings And Interactions with Cells

**Fig. 10:**
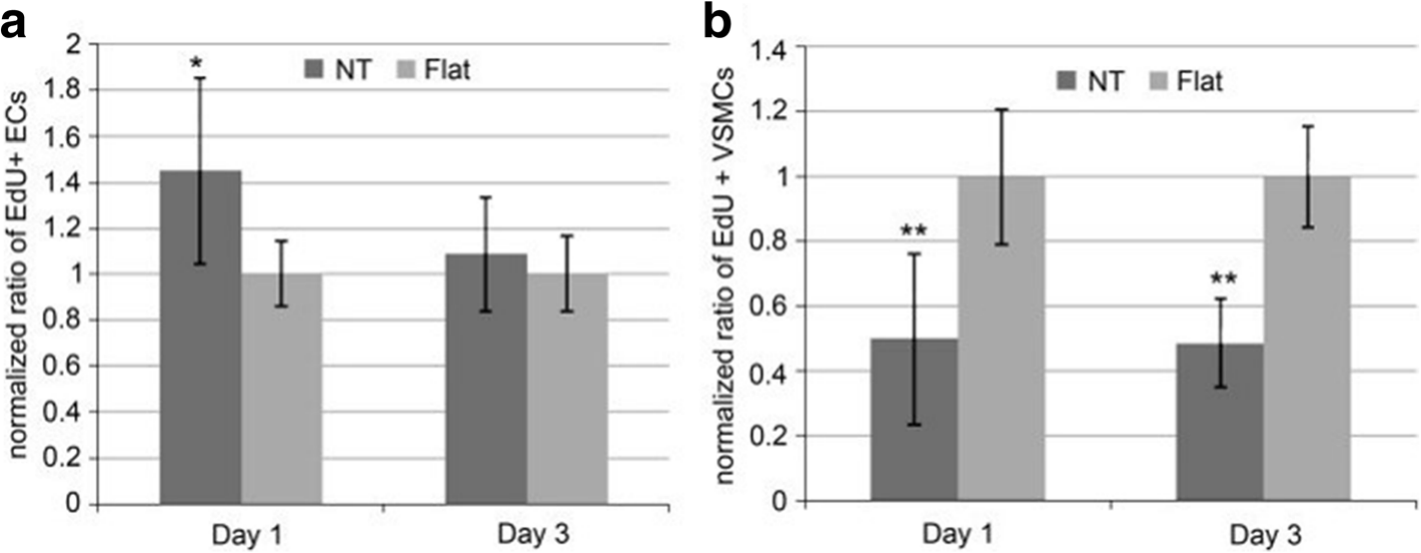
Ratio of EdU positive **a** ECs and **b** VSMCs on flat or nanotube substrate. It is normalized by the average proportion of positive cells on flat surfaces on day 1 and 3. Data is presented as average ± standard deviation. *p < 0.05, **p < 0.01 versus same day flat control, *n* = 6 reproduced from ref. [161]

A number of in vitro and in vivo studies have demonstrated that MSCs, osteoblasts and osteoclasts show size-selective response which means the effect of size holds an important position in cell interaction where the optimized size for cell adhesion, proliferation, growth, and differentiation is ranging from 15 to 100 nm [153, 157, 158]. Particularly, it was demonstrated that the TiO_2_ nanotubes with a diameter of 70 nm was the optimal nanoscale geometry for the osteogenic differentiation of human adipose-derived stem cells (hASCs) [159]. Smith et al. reported increased dermal fibroblasts and decreased epidermal keratinocyte adhesion, proliferation, and differentiation on TiO_2_ nanotube arrays (diameter 70–90 nm, length 1–1.5 μm) [160]. As shown in Fig. 10, Peng et al. found that nanotubular surface preferentially promoted proliferation and function in endothelial cells (EC) while decreased in vascular smooth muscle cell (VSMC) by measuring EdU, a thymidine analog which is incorporated by proliferating cells [161]. Furthermore, it is pointed out that surface wettability of the TiO_2_ nanotube layers is recognized as a critical factor for cell behavior which can be adjusted by changing the diameter of the nanotubes. That is to say, water contact angles can be altered without changing the surface chemistry [158]. To get further understanding of the effect of TiO_2_ nanotube layers to bone-forming cells as well as stem cells response, Park et al. seeded green fluorescent protein-labeled rat MSCs on TiO_2_ nanotube layers with six different diameters (15, 20, 30, 50, 70, and 100 nm), resulting in cell activity that is sensitive to nanoscale surface topography with a maximum in cell activity obtained for tube diameters of approximately 15–30 nm. Such lateral spacing exactly corresponds to the predicted lateral spacing of integrin receptors in focal contacts on the extracellular matrix, forcing clustering of integrins into the closest packing, resulting in optimal integrin activation. While tube diameters larger than 50 nm, severely impaired cell spreading, adhesion, and spacing of 100 nm may lead to the cell apoptosis [94]. Besides adjusting the size of the nanotubes, surface modification loaded with bioactive factors should be highlighted, in which case biomedical properties can be further optimized. In the case of bone implants, hydroxyapatite (HA) formation is important for osseointegration. Recent works have shown hydroxyapatite nanocrystalline coating onto the nanotubular TiO_2_ results in further enhanced osseointegration with strong adhesion and bond strength, and a drastic enhancement of deposition rate is observed [162, 163]. Nanotubular TiO_2_ surface can greatly enhance the natural apatite growth rate in simulated body fluid (SBF) compared with flat surfaces [10, 164]. The alkaline-treated TiO_2_ nanotubes with NaOH solutions are more bioactive in SBF, where sodium titanate can significantly accelerate nucleation and the growth of HA formation presenting a well-adhered bioactive surface layer on Ti due to its larger surface area and promoted mechanical interlocking between HA and TiO_2_ nanotubes [165, 166]. Electrodeposited with hydroxyapatite, higher adhesion of TiO_2_ nanotubes has been described in the literature by means of adhesive tape test and the live/dead cell staining study which is essential for early bone formation [166]. The results also showed that at the length of 560 nm the highest adhesion of HA surface on the nanotubes is observed. Also the nanotube surface can indeed strengthen Collagen type I expression in vivo experiment which is considered to be a basic initial bone matrix protein in bone formation [167]. Moreover, annealing of the amorphous nanotubes to anatase or a mixture of anatase and rutile was found to be an important factor in the apatite formation process [164].

### Drug Delivery and Antibacterial Ability

Furthermore, the tubular nature of TiO_2_ in biomedical devices may be exploited as gene and drug delivery carriers with living matter due to its high surface area, controllable pore, and self-ordered structure [1, 15]. When the orthopedic bioimplant is placed into the bone defect, persistent and chronic infection is one of the most common and serious complications associated with biomedical implantation [16, 168]. Certain dimension and crystallinity may be useful to prevent bacteria adhesion and promote bone formation. The thermal annealing has decreased the number of bacteria adhering to the Ti surface. It could be in part because heat treatment removes the fluorine content which has a tendency to attract bacteria. The research also indicates that nanotubes with 60 or 80 nm in diameter decrease the number of live bacteria as compared to lower diameter (20 or 40 nm) nanotubes [169, 170].

Bauer et al. loaded epidermal growth factor (EGF) and bone morphogenetic protein-2(BMP-2) onto the TiO_2_ nanotubes surface by covalent attachment. They observed positive influence on the behavior of MSCs on 100-nm nanotube arrays where cell count was at much higher levels compared to the untreated one [171]. Lately, titania nanotubes loaded with antibiotics contribute to suppressing bacterial infections. As gentamicin sulphate (GS) is mostly widely used with highly water solubility, Feng et al. loaded titania nanotubes with GS through physical adsorption and cyclic loading which can treat many types of bacterial infections [172]. Zhang et al. fabricated titania nanotubes loaded with vancomycin to investigate the increasing biocompatibility and obvious antibacterial effect on *Staphylococcus aureus* [173]. However, systemic antibiotics in clinical will bring many side effects. The release of antibiotics from the nanotubes is too fast to maintain the long-term antibacterial ability, and the use of antibiotics may develop resistant strains [126, 168, 174]. Ensuring a constant release rate becomes a crucial but difficult part in the field of drug delivery. In strategies like surface modification, controlling the dimension of nanotube arrays, biodegradable polymer coating have been employed to solve the issue [21]. Drug release of several drugs such as antibiotics or growth factors from titania nanotube arrays can be adjusted by varying their diameters and lengths [152, 175, 176]. Feng et al. covered a thin film comprising a mixture of GS and chitosan on GS-loaded titania nanotubes and showed a controlled release of the drug providing sustained release effects to a certain extent [172]. Titania nanotube arrays as drug nanoreservoirs on Ti surface for loading of BMP-2 were fabricated by Hu et al. and then further covered with gelatin/chitosan multilayers to control the release of the functional molecule meanwhile maintain the bioactivity for over 120 h via a spin-assisted layer-by-layer assembly technique which is mainly based on electrostatic interactions between polyanions and polycations as well as promote osteoblastic differentiation of MSCs [177]. Lai et al. successfully fabricated Chi/Gel multilayer on melatonin-loaded TiO_2_ nanotube arrays to control the sustained release of melatonin and promote the osteogenic differentiation of mesenchymal stem cells [178]. Karan et al. synthesized titania nanotubes loaded with the water-insoluble anti-inflammatory drug indomethacin and modified lactic-co-glycolic acid on surface as a polymer film in order to extend the drug release time of titania nanotubes and produce favorable bone cell adhesion properties, with reduced burst release (from 77 to > 20%) and extended overall release from 4 days to more than 30 days [152]. As previous study reported that surface treatment of implants with *N*-acetyl cysteine (NAC) may reduce implant-induced inflammation and promote faster bone regeneration [179], Lee et al. examined the feasibility of *N*-acetyl cysteine-loaded titania nanotubes as a potential drug delivery system onto an implant surface, and the data indicates the enhanced osseointegration and the value of the small animal model in assessing diverse biological responses to dental implants. Besides, TiO_2_ nanotube arrays are suitable for loading inorganic agents like Ag, Sr, and Zn to obtain long-term antibacterial ability and osseointegration [126, 180–182]. Ag nanoparticles have been incorporated into TiO_2_ nanotube arrays previously with satisfactory small possibility to develop resistant strains, a broad-spectrum antibacterial property, low cytotoxicity, and good stability by immersion in a silver nitrate solution followed by ultraviolet light radiation [126]. Zhang et al. demonstrated that a series of porous TiO_2_ coatings with different concentrations of silver had significant inhibition effect on *Escherichia coli* and *Staphylococcus aureus*. Besides, only with the optimum amount of silver can the coatings retain the antibacterial effect but without any measurable cytotoxicity to cells [183]. Due to cytotoxicity observed by the excessive release of Ag^+^ subsequently, titania nanotube arrays with Ag_2_O nanoparticles embedded in the wall are prepared on Ti by TiAg magnetron sputtering and anodization in order to get slower and more controllable silver ion release [184]. That is because the TiO_2_ barrier is surrounded thereby minimizing the cytotoxicity induced by burst or large Ag^+^ release.

**Fig. 11:**
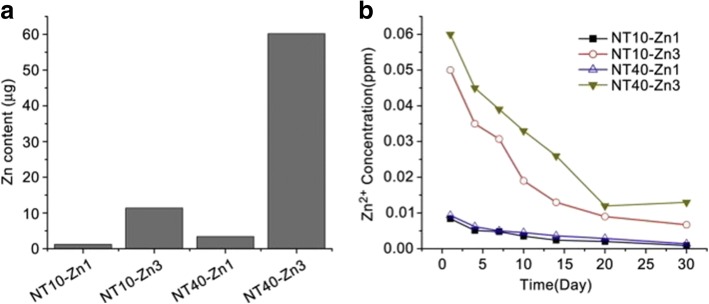
**a** Total amounts of Zn incorporated into the NT-Zn samples for the 1 cm^2^ coatings and **b** non-cumulative Zn release profiles from NT-Zn into PBS. Reproduced from ref. [181]

**Fig. 12:**
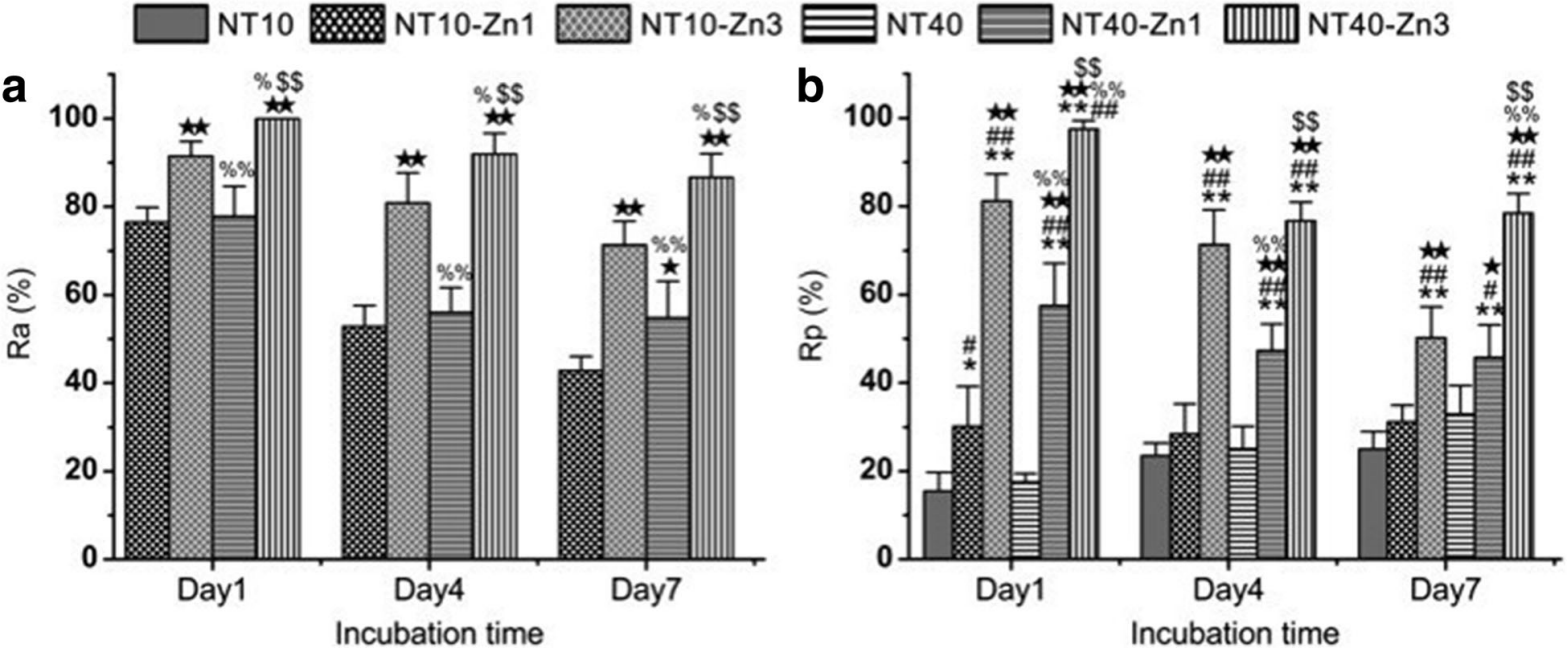
**a** Antibacterial rates versus adherent bacteria on the specimen (Ra) and **b** antibacterial rates against planktonic bacteria in the medium (Rp) *, **p < 0.05 and 0.01 vs NT10; ^#^, ^##^*p* < 0.05 and 0.01 vs NT40; ^★^, ^★★^*p* < 0.05 and 0.01 vs NT10-Zn1; ^%^, ^%%^*p* < 0.05 and 0.01 vs NT10-Zn3; ^$^, ^$$^*p* < 0.05 and 0.01 vs NT40-Zn1. Reproduced from ref. [181]

Similar to Ag, Zn possesses antibacterial and anti-inflammation properties, and osteogenesis induction [185–187]. Huo et al. produced anodic TiO_2_ nanotube arrays at 10 V and 40 V (NT10 and NT40) incorporated with Zn by hydrothermal treatment at 200 °C for 1 and 3 h (NT10-Zn1, NT10-Zn3, NT40-Zn1, and NT40-Zn3) in Zn containing solutions, followed by annealing at 450 °C for 3 h in air. NT40-Zn3 has the largest Zn loading capacity and releases more Zn compared with other samples. The amounts of Zn released diminish gradually with time and nearly no Zn can be detected 1 month later except sample NT40-Zn3 (Fig. 11). The NT-Zn samples present different antibacterial ability. It is evident that NT40-Zn3 and NT10-Zn3 effectively kill more adherent bacteria as well as surrounding planktonic bacteria in the early stage. Figure 12a describes a synergistic effect of both released and surface incorporated Zn while Fig. 12b explains the effect of the released Zn [181].

## Conclusions

This review presents the historical developments and traditional formation mechanism of titania nanotube arrays grown by electrochemical anodization as well as the approaches to influence and modify morphology in order to improve their performances. We also focus on current efforts toward TiO_2_ nanotubes applications in biomedical directions. Those steady progresses have demonstrated that TiO_2_ nanotubes are playing and will continue to play an important role in material science, but there are still some aspects needed to be further improved.

1. The synthesis of TiO_2_ nanotube arrays is already comparatively mature so far in fact, but how to simplify the technology for the purpose of large-scale production in industry with extending practical operability and how to precisely control nanotube geometry efficiently by varying the anodic parameters so as to obtain optimized properties have yet to be further investigated.
2. The formation mechanisms of anodic TiO_2_ nanotubes have gradually become a hotspot of research due to their unique structure and excellent performances but the exact mechanism remains controversial. Conventional FAD explains the growth process and the porous structure of TiO_2_ nanotubes, but the combination of viscous flow model and growth model of two currents can give a comprehensive explanation to the growth process. Notably, the validity of oxygen evolution resulting from electronic current has much room for investigation.
3. Modification is key for improving performances of titania nanotube arrays. Thus, we need to explore more methods for modification and take full advantage of the self-organized nanostructure. Through self-assembling inorganic, organic, metallic, and magnetic nanoparticles into or onto the tubes as nanocomposites with broad spectral response to visible light, high quantum efficiency, and stabilizing properties, applications could be widened. Currently, ALD appears to be an option to coat the titania nanotube layers homogenously and precisely from the bottom to the tube mouth, resulting in many advanced functionalities of the newly prepared nanotube layers. Nevertheless, further optimization of the ALD process toward coatings and inner fillings is demanded.
4. TiO_2_ nanotube researches in biomedical directions are still in their infancy and have a long distance to go in clinical use. The biological reaction between cells and titania nanotubes has to develop from cellular level to molecular level and from morphological changes to molecular alterations. It has been shown that adhesion, spreading, and growth of osteoblast and mesenchymal stem cells strongly depends on nanotube diameter, so the regularity and principle of this phenomenon as well as other factors affecting cells’ behaviors need to be further explored.

## Abbreviations

ALD: Atomic layer deposition
BMP-2: Bone morphogenetic protein-2
DMSO: Dimethyl sulfoxide
DSSCs: Dye-sensitized solar cells
EBSD: Electron Backscatter Diffraction
EC: Endothelial cells
EdU: A thymidine analog
EGF: Epidermal growth factor
FAD: Conventional field-assisted dissolution
FE-SEM: Field-emission scanning electron microscopy
Fe-TNTs: Fe-doped TiO2 nanotubes
FIB: Focused ion beam
GO: Graphene oxide
GS: Gentamicin sulphate
HA: Hydroxyapatite
hASCs: Adipose-derived stem cells
IPCE: Incident photon-to-electron conversion efficiency
MSCs: Mesenchymal stem cells
NAC: *N*-Acetyl cysteine
N-TNT: N-doped TiO2 nanotubes
PEC: Photoelectriochemical
SBF: Simulated body fluid
UV: Ultraviolet
VSMC: Vascular smooth muscle cell
